# The history and advances in cancer immunotherapy: understanding the characteristics of tumor-infiltrating immune cells and their therapeutic implications

**DOI:** 10.1038/s41423-020-0488-6

**Published:** 2020-07-01

**Authors:** Yuanyuan Zhang, Zemin Zhang

**Affiliations:** 1 Beijing Advanced Innovation Center for Genomics, Peking-Tsinghua Center for Life Sciences, Academy for Advanced Interdisciplinary Studies, Peking University, 100871 Beijing, China; 2 BIOPIC and School of Life Sciences, Peking University, 100871 Beijing, China

**Keywords:** Immunotherapy, Tumor microenvironment, Single-cell technologies, Tumor-infiltrating immune cells, Phenotypic diversities, Cancer microenvironment, Tumour immunology

## Abstract

Immunotherapy has revolutionized cancer treatment and rejuvenated the field of tumor immunology. Several types of immunotherapy, including adoptive cell transfer (ACT) and immune checkpoint inhibitors (ICIs), have obtained durable clinical responses, but their efficacies vary, and only subsets of cancer patients can benefit from them. Immune infiltrates in the tumor microenvironment (TME) have been shown to play a key role in tumor development and will affect the clinical outcomes of cancer patients. Comprehensive profiling of tumor-infiltrating immune cells would shed light on the mechanisms of cancer–immune evasion, thus providing opportunities for the development of novel therapeutic strategies. However, the highly heterogeneous and dynamic nature of the TME impedes the precise dissection of intratumoral immune cells. With recent advances in single-cell technologies such as single-cell RNA sequencing (scRNA-seq) and mass cytometry, systematic interrogation of the TME is feasible and will provide insights into the functional diversities of tumor-infiltrating immune cells. In this review, we outline the recent progress in cancer immunotherapy, particularly by focusing on landmark studies and the recent single-cell characterization of tumor-associated immune cells, and we summarize the phenotypic diversities of intratumoral immune cells and their connections with cancer immunotherapy. We believe such a review could strengthen our understanding of the progress in cancer immunotherapy, facilitate the elucidation of immune cell modulation in tumor progression, and thus guide the development of novel immunotherapies for cancer treatment.

## Introduction

Cancer is a disease of the genome, and it is characterized by a genomic instability in which numerous point mutations accumulate and structural alterations occur in the process of tumor progression.^1,2^ Such genomic variations could give rise to tumor antigens, which could be recognized by the immune system as nonself and elicit cellular immune responses.^3,4^ The immune system plays an essential role in immunosurveillance,^4,5^ as immune cells of the adaptive and innate immune systems infiltrate into the tumor microenvironment (TME) and contribute to the modulation of tumor progression.^6,7^ Innate immune cells, composed of natural killer (NK) cells, eosinophils, basophils, and phagocytic cells, including mast cells, neutrophils, monocytes, macrophages, and dendritic cells (DCs), participate in tumor suppression either by directly killing tumor cells or by triggering adaptive immune responses.^8–10^ The adaptive immune system functions with lymphocytes, including B cells and T cells, among which B cells play a major role in humoral immune responses, whereas T cells are involved in cell-mediated immune responses.^5,11,12^

Effective immune responses could either eradicate malignant cells or impair their phenotypes and functions.^3^ However, cancer cells have evolved multiple mechanisms, such as defects in antigen presentation machinery, the upregulation of negative regulatory pathways, and the recruitment of immunosuppressive cell populations,^13–17^ to escape immune surveillance, resulting in the impeded effector function of immune cells and the abrogation of antitumor immune responses.

Immunotherapy, aiming to boost natural defenses to eliminate malignant cells, is a monumental breakthrough for cancer treatment and has revolutionized the field of oncology. Although the idea of unleashing the host immune system to eradicate cancer could trace back to a century ago,^18,19^ significant advances have been achieved in recent basic and clinical investigations. Multiple cancer types have shown sustained clinical responses to immunotherapy,^20–25^ albeit with limited response rates and unclear underlying mechanisms.^26^ Immune cells are the cellular underpinnings of immunotherapy; thus, understanding the immune infiltrates in the TME is the key to improving responsive rates and developing new therapeutic strategies for cancer treatment with immunotherapy. Although the tumor–immune ecosystem is highly complex and comprises a heterogeneous collection of cells, single-cell technologies have emerged as powerful tools for the dissection of the TME.^27^ Although tremendous efforts have been devoted to T-cell characterizations, other immune cells of the innate and adaptive immune systems, including DCs, macrophages, NK cells, and B cells, have also been shown to contribute to tumor progression and immunotherapy responses. In this review, we will outline the major categories of cancer immunotherapy and the history of their development, as well as the recent findings on tumor-infiltrating immune cells in human cancers, their connections with immunotherapy and potential clinical applications.

## The rise of major categories of immunotherapy

**Fig. 1:**
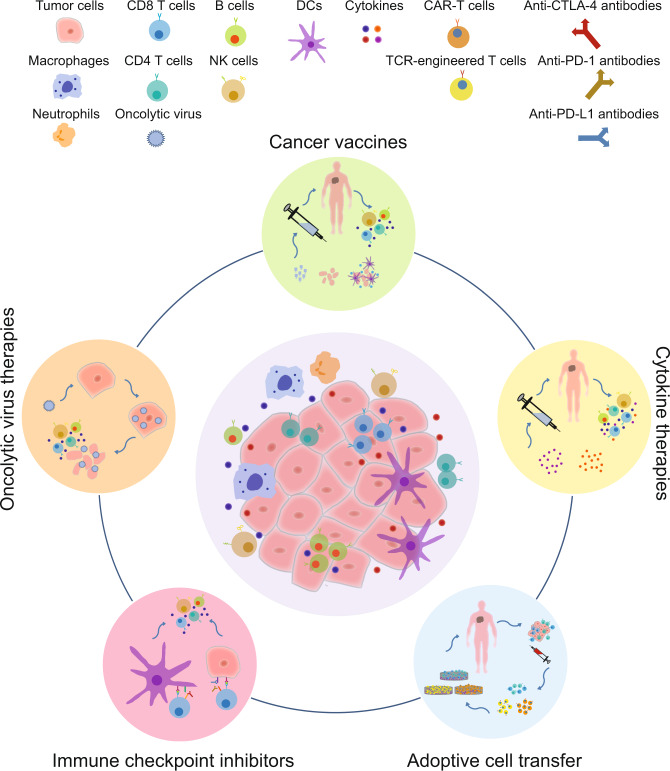
The major categories of immunotherapy. Different forms of cancer immunotherapy, including oncolytic virus therapies, cancer vaccines, cytokine therapies, adoptive cell transfer, and immune checkpoint inhibitors, have evolved and shown promise in clinical practice. The basic principles of each strategy and the corresponding cellular and molecular underpinnings involved in each step are depicted. DCs dendritic cells, NK natural killer, TCR T-cell receptor, CAT-T chimeric antigen receptor T-cell

Here, we briefly review the historical studies that led to the development of several major types of immunotherapy applied in cancer treatment (Fig. 1).

### Oncolytic virus therapies

For more than a century, traditional immunotherapy has approached cancer by harnessing bacterial or viral infection to enhance immune responses. As early as 1863, Virchow first discovered the connection between tumors and inflammation after observing that neoplastic tissues were often decorated with leukocytes of the immune system.^28^ The earliest case of cancer immunotherapy can be traced back to 1891, when William Coley, the father of immunotherapy, first attempted to leverage the immune system to treat cancer after noticing that mixtures of live and inactivated *Streptococcus pyogenes* and *Serratia marcescens* could cause tumor regression in sarcoma patients.^29,30^ Although such a pioneering strategy provided a proof of concept for treating cancer by the utilization of the immune system, the unknown mechanisms of action and the potential infection risks hindered its further progress. Decades later, oncolytic virus therapies were invented, which leverage genetically modified viruses to infect tumor cells, and thus stimulate a proinflammatory environment to augment systemic antitumor immunity.^31,32^ With advances in genetic engineering and virus transformation technologies, oncolytic virus therapies have made much progress in recent years. In particular, talimogene laherparepvec (T-Vec), also known as Imlygic, a genetically modified *herpes simplex virus*, demonstrates impressive clinical benefits for patients with advanced melanoma and has been approved for the treatment of unresectable metastatic melanoma.^33^

### Cancer vaccines

Cancer vaccines utilize tumor-specific antigens to trigger T-cell-mediated antitumor immune responses. Pivotal studies came from the identification of MZ2-E and MZ2-D, both of which are melanoma-derived antigens encoded by the MAGE (melanoma-associated antigen) gene family that could be recognized by cytotoxic T cells to trigger antitumor immune responses.^34,35^ Simultaneously, another human melanoma antigen, gpl00, was proven to be associated with tumor rejection in vivo by inducing immune responses mediated by tumor-infiltrating lymphocytes (TILs) in melanoma patients.^36^ These findings paved the way for utilizing tumor antigens as vaccines in cancer immunotherapy. Aside from tumor antigens, DC-based vaccination also showed significant clinical outcomes. DCs are the best equipped antigen-presenting cells (APCs) and play critical roles in eliciting antitumor immunity.^37^ Specifically, after activation by tumor antigens, DCs can internalize, process, and subsequently present the processed epitopes to T cells and induce cytotoxic T lymphocyte (CTL) immune responses.^37^ Due to their proficiency at antigen presentation, DCs are leveraged in DC-based vaccines, which involve the reinfusion of isolated DCs pulsed with tumor antigens or tumor cell lysates and stimulated with a defined maturation cocktail ex vivo.^38^ One representative example is sipuleucel-T, a DC-based immunotherapy that has been approved for the treatment of advanced prostate cancer.^39^ Furthermore, whole tumor cells can also be utilized to evoke spontaneous immune responses. GVAX, a cancer vaccine composed of autologous tumor cells genetically modified to secrete granulocyte-macrophage colony-stimulating factor, was developed^40^ and showed promise in augmenting tumor-specific immune responses in multiple cancer types.^41–43^ These advances underline the importance of tumor vaccines in clinical applications for cancer treatment.

### Cytokine therapies

Functioning as messengers to orchestrate cellular interactions and communications of the immune system, cytokines are released by immune and nonimmune cells in response to cellular stresses such as infection, inflammation, and tumorigenesis.^44^ The secreted cytokines enable the rapid propagation of immune signaling in a complex yet efficient manner, and thus could generate potent and coordinated immune responses to target antigens.^44,45^ The potential application of cytokines in cancer treatment benefits from the identification of interleukin 2 (IL-2) in 1976.^46^ IL-2, initially named T-cell growth factor, has the ability to expand T cells in vitro and in vivo, and thus exerts immune-stimulatory properties.^47–49^ As a typical instance of cytokine therapies, the administration of large doses of IL-2 in clinical applications could lead to cancer regressions in patients with metastatic cancer.^50,51^ In addition to IL-2, interferon-alpha (IFN-α) also serves as a classic therapeutic cytokine in cancer treatment. Interferons (IFNs) comprise a large family of cytokines, among which IFN-α, a pleiotropic cytokine of type I IFN, is a critical determinant of the efficacy of antitumor immunity.^52^ IFN-α plays multifaceted roles in tumor control, including directly eradicating tumor cells through inducing senescence and apoptosis and boosting effective antitumor immune responses through the stimulation of DC maturation and the enhancement of T-cell cytotoxicity.^52^ Clinical studies have proven the therapeutic role of IFN-α at high dosages in chronic myeloid leukemia and melanoma.^53,54^ Despite clinical benefits, poor tolerability and severe toxicity hamper further applications of these cytokines as monotherapies, but cytokines are still being investigated in combination with other immunotherapies, such as adoptive cell transfer (ACT) therapy, to circumvent such impediments.

### Adoptive cell transfer

ACT therapies utilize autologous immune cells, in particular T cells, which are isolated or genetically engineered, ex vivo expanded, and reinfused back into patients to eliminate cancer cells and have shown sustained clinical efficacy.^55–57^ Rosenberg et al. demonstrated that large doses of IL-2 accompanied by autologous lymphokine-activated killer cells were effective when administered to patients with metastatic cancers.^51^ After this first trial of successful adoptive immunotherapy, the team subsequently improved this approach with TILs and demonstrated that the adoptive transfer of TILs expanded in IL-2 showed more therapeutic potency.^58,59^ These studies provide a rationale for the use of TILs to treat advanced human cancers.

Hereafter, the adoptive transfer of highly selected tumor-reactive T cells against overexpressed, self-derived differentiation antigens to patients with metastatic melanoma led to the persistent clonal repopulation of T cells in cancer patients,^60^ enlightening the use of genetically manipulated T cells that target specific neoantigens in adoptive transfer. Currently, two types of genetically modified T cells, chimeric antigen receptor (CAR)-T cells and T-cell receptor (TCR)-engineered T cells, have been invented for adoptive transfer and have achieved substantial advances in the treatment of malignant tumors.

CAR-T-cell therapies utilize antibody fragments to recognize specific antigens expressed on the surface of cancer cells. The first generation of CAR-T cells involved genetically modified T cells with antibody specificity by expressing immunoglobulin-TCR chimeric molecules as functional receptors.^61^ However, these CAR-T cells were unable to persist in the body until 1998, when Maher et al. established a new generation of CAR-T cells by introducing costimulatory molecules such as CD28 into the engineered CARs to allow modified T cells to persist and remain active in the body.^62,63^ They subsequently demonstrated that CD19-specific CD28/CD3-zeta dual-signaling CAR-modified T cells could induce molecular remissions in adult acute lymphoblastic leukemia.^64^ In addition, other molecules have been examined for their efficacies when conjugated in CARs, and Porter et al. demonstrated that autologous T cells genetically modified to target B-cell antigen CD19 by expressing anti-CD19 linked to CD3-zeta and 4–1BB signaling domains could generate potent CD19-specific immune responses in chronic lymphocytic leukemia (CLL) patients.^65^ These findings shed light on the promising antitumor efficacy of CAR-T therapies in human cancers. TCR-engineered T-cell or TCR-T therapy was first reported by Clay et al., who demonstrated that TCR gene transfer to peripheral blood lymphocytes (PBLs) derived from melanoma patients could generate effector T cells with antitumor reactivity in vitro.^66^ The clinical potential of such a therapy was subsequently confirmed in metastatic melanoma patients with regressed tumors when treated with TCR-engineered T cells.^67^ Notably, the canonical cancer-testis antigen NY-ESO-1, aberrantly expressed in cancer cells,^68,69^ has been targeted using genetically modified TCR-T cells, which have mediated sustained antigen-specific antitumor effects and finally led to tumor regression.^57,70^ Thus, both CAR-T-cell and TCR-T-cell therapies have achieved substantial advances in cancer treatment and have generated encouraging clinical outcomes.

### Immune checkpoint inhibitors

Despite the monumental progress in ACT therapies, a new class of monoclonal antibodies (mAbs),^71^ immune checkpoint inhibitors (ICIs), are now entering medical practice and have become one of the most important immunotherapies. Immune checkpoints are molecules of coinhibitory signaling pathways that act to maintain immune tolerance, yet they are often utilized by cancer cells to evade immunosurveillance.^72,73^ ICIs are designed to reinvigorate antitumor immune responses by interrupting coinhibitory signaling pathways and to promote immune-mediated elimination of malignant cells.^74,75^ The most widely used targets for ICIs are cytotoxic T lymphocyte-associated molecule-4 (CTLA-4), programmed cell death receptor-1 (PD-1), and programmed cell death ligand-1 (PD-L1).

CTLA-4 is a coinhibitory molecule expressed on T cells and functions to negatively regulate T-cell activation.^76,77^ One pioneering study demonstrated that blocking CTLA-4 with antibodies could induce effective immune responses and lead to tumor regression,^78^ opening the era of utilizing antibodies to release the brakes of immune cells to reinforce antitumor immune responses.^78,79^ After clinical trials and efficacy evaluations,^20,80^ ipilimumab, a CTLA-4 mAb, became the first ICI approved for cancer treatment due to its ability to enhance T-cell activation and induce durable responses.^20,81^ In parallel, PD-1 was discovered to be expressed on the surface of T cells and was originally thought to be involved in programmed cell death,^82^ yet, later, it was proven to act as a negative regulator of immune responses.^83,84^ However, the regulatory mechanisms of PD-1 remained elusive until the discovery of its ligand, PD-L1,^85^ which is expressed in normal tissues and regulates immune tolerance by suppressing TCR-mediated lymphocyte proliferation and cytokine secretion when binding with PD-1.^86,87^ Tumor cells, however, also abnormally express PD-L1 to escape immune surveillance.^88,89^ Studies have shown that the inhibition of PD-1 or PD-L1 could reinvigorate the cytotoxic ability of T cells and induce tumor regression,^90,91^ suggesting that PD-1 or PD-L1 could serve as therapeutic targets. Indeed, the blockade of the PD-1 pathway has achieved remarkable clinical outcomes, and antibodies targeting PD-1 or PD-L1 have been approved for the treatment of multiple cancers.^20,92^

## Tumor-infiltrating immune cells and their associations with immunotherapies

The success of cancer immunotherapy, such as ACT and ICI therapies, has demonstrated that immune cells, particularly T cells, can be harnessed to eliminate tumor cells. Despite the sustained clinical efficacy, however, only a fraction of cancer patients benefit from them.^93^ As a major component of the TME, immune infiltrates have been proven to contribute to tumor progression and immunotherapy responses.^94^ Therefore, a better understanding of both innate and adaptive immune cells in the TME is essential for deciphering the mechanisms of immunotherapies, defining predictive biomarkers, and identifying novel therapeutic targets.

### T cells

T cells have become the focus of tumor immunology due to their potent tumor-killing capability.^95,96^ The function of T cells is initiated through the engagement of TCRs with short peptides of tumor antigens presented by major histocompatibility complex (MHC) molecules or human leukocyte antigen. TCRs are produced by genetic rearrangements involving a large number of random recombinations of TCR gene segments, the process of which could generate diverse TCR repertoires, endowing T cells with diversity and specificity.^97,98^ TILs play a pivotal role in effective antitumor immunity, and different types of T cells, including cytotoxic T cells, T helper (T_H_) cells, and regulatory T cells (Tregs), are involved in T-cell-mediated immune responses within the tumor environment.^99^

CTLs are the key effector cells, functioning with cytotoxic molecules such as granzymes and perforin.^100^ Although studies have shown that the presence of TILs, in particular CTLs, is positively correlated with patient survival in multiple cancers,^101,102^ CTLs that infiltrate tumor sites often fail to control tumor growth due to exhaustion or dysfunction sculpted by the immunosuppressive TME.^94,103–105^ T-cell exhaustion, characterized by the upregulation of PD-1 and other inhibitory molecules, was originally described in murine models of chronic lymphocytic choriomeningitis virus infection^106,107^ and proven to be prevalent in human cancers.^107^ For instance, Thommen et al. analyzed the properties of three populations of intratumoral CD8^+^ TILs with different levels of PD-1 expression from non-small cell lung cancer (NSCLC) patients and found that TILs with high PD-1 expression were exhausted yet predictive for responses to anti-PD-1 treatment in NSCLC patients.^108^ Such findings, along with the impressive clinical efficacy of ICIs,^109–111^ highlight the importance of intervening in T-cell dysfunction in cancer treatment.

CD4 T cells comprise T_H_ cells and Tregs. T_H_ cells contribute to antitumor immunity either by helping CD8 effector T cells^112^ or by acting as cytotoxic T cells to directly eliminate tumor cells.^113,114^ In contrast, Tregs, which are indispensable for maintaining homeostasis,^115^ orchestrate antitumor immunity by directly undermining T-cell function via immunosuppressive soluble factors, as well as by indirectly impeding T-cell activation via CTLA-4-mediated inhibition of costimulatory signals of APCs.^116,117^ Notably, despite the effect of blocking negative signaling to strengthen T-cell priming, anti-CTLA-4 antibodies could also induce Treg depletion,^118,119^ indicating the complex mechanisms of ICIs contributing to antitumor immunity.

### B cells

B cells are humoral immune cells that function in the humoral immunity of the adaptive immune system. In response to infected cells or tumor cells, B cells differentiate into memory B cells or plasma cells, the latter of which can secrete immunoglobulins (Igs), also known as antibodies, to bind and neutralize target antigens.^120^ Notably, the activation of B cells involves the interaction of antigens with the B-cell receptor (BCR), a membrane-bound form of Ig (mIg) endowing B cells with antigen specificity. Each B-cell harbors a unique BCR derived from a highly diverse pool of the BCR repertoire generated from the random rearrangement of the Ig gene segments.^120^ The BCR repertoire carries diverse antigen specificities, and upon antigen encounter, the selected BCR could be further modified by class-switch recombination and somatic hypermutation within the germinal center, resulting in optimized antibodies against target antigens.^121,122^

Although B cells play crucial roles in humoral immunity by antibody production, they also contribute to cellular immunity by serving as APCs to enhance T-cell-mediated immunity and by modulating immune responses through cytokines or regulatory B cells.^123,124^ Moreover, B cells help maintain secondary lymphoid organ architecture and facilitate the formation of tertiary lymphoid structures (TLSs), highly organized structures composed of aggregates of immune cells such as T cells, B cells, and follicular DCs, at sites of chronic inflammation and tumors.^125^ TLSs are particularly important for the recruitment and local activation of B cells and T cells, and thus contribute to long-term immunity.^125,126^

Because of such diverse functions in both humoral and cellular immunity, B cells exhibit phenotypic diversity in antitumor immunity.^127^ Tumor-infiltrating B cells (TIBs) have been demonstrated to promote tumor progression by inhibiting T-cell-mediated immune responses by secreting soluble mediators that impel the proangiogenic and protumorigenic functions of myeloid cells or by producing factors that facilitate signal transduction in cancer cells.^128–130^ However, in contrast to the cancer-promoting effects, accumulating studies have shown that B cells function in antitumor immunity and could favor patient prognosis. CD20^+^ TIBs have been shown to correlate with favorable prognosis in patients with NSCLC and ovarian cancer, possibly by acting as APCs to augment cytolytic T-cell responses.^131,132^ Notably, Cabrita et al. revealed that the formation of TLSs, as well as the cooccurrence of CD20^+^ B cells and CD8^+^ T cells in tumors, was associated with improved survival for patients with metastatic melanomas and could predict clinical outcomes of ICIs.^133^ In line with these observations, B cells have been implicated in immunotherapy responses. Hollern et al. uncovered that ICIs could induce the activation of T follicular helper cells and B cells, and activated B cells could facilitate antitumor responses by secreting antibodies and by activating T cells through antigen presentation in high mutation burden mouse models of triple-negative breast cancer.^134^ Concordant with these findings in the murine study, clinical investigations also underscore the importance of B cells, accompanied by TLSs, in cancer immunotherapy. Jahrsdörfer et al. illustrated that B cells could produce granzyme B and obtained cytotoxic capability after IL-21-based activation in B-CLL.^135^ In addition, Petitprez et al. found that a subtype of soft-tissue sarcoma patients, characterized by the presence of TLSs containing B cells and other immune cells, had improved survival and a high response rate to PD-1 blockade.^136^ Consistently, Helmink et al. showed that CD20^+^ B cells were colocalized with T cells in TLSs of tumors of patients with metastatic melanoma and metastatic renal cell carcinoma (RCC) responsive to ICI treatment.^125^ In addition, they further identified significant clonal expansion and BCR diversity in responders, providing insights into the pivotal roles of B cells and TLSs in cancer immunotherapy.

Therefore, these findings pinpoint the crucial roles of B cells in antitumor immune regulation and indicate that B cells and TLSs have significant applications for cancer treatment, although further investigations are needed to illuminate the mechanisms of B-cell-mediated responses to immunotherapies.

### NK cells

NK cells are prototypical innate lymphoid cells that exert cytotoxic functions without MHC specificity, and thus complement the MHC-restricted tumor lysis mediated by cytotoxic T cells.^137,138^ NK cells directly eradicate tumor cells through cytolytic granules and cooperate with other immune cells through proinflammatory cytokines and chemokines.^138–140^ Importantly, the activation of NK cells is mediated by the combined action of activating and inhibitory receptors expressed on the NK cell surface. Specifically, inhibitory receptors interact with MHC class I molecules expressed on normal cells and contribute to the self-tolerance of NK cells, while activating receptors sense the signals of cellular stress associated with viral infection or tumorigenesis when virus-infected cells or tumor cells lose MHC class I expression, leading to NK activation and effector function.^139^ However, emerging studies indicated that NK cells showed impeded function with reduced cytotoxic activity and altered expression of proinflammatory cytokines in the TME.^139^ Böttcher et al. discovered that NK cells in tumors could recruit cDC1 cells into the TME to facilitate antitumor immunity, while tumor cells could produce prostaglandin E2 to impair NK cell functions, leading to immune evasion.^140^ Therefore, NK cells could serve as possible targets as well.

Several NK-based immunotherapies have been explored, including adoptive transfer of autologous NK cells, which refers to the transfusion of ex vivo activated and expanded NK cells into patients;^141^ CAR-NK cell therapies, which involve the transfusion of engineered NK cells expressing CARs against a specific tumor antigen;^142^ cytokine therapies, which involve the infusion of specific cytokines to augment NK cell activity;^143^ and mAb-based therapies, referring to the delivery of antibodies to block inhibitory receptors on NK cells.^144^

Analogous to ICIs, which block inhibitory pathways in T cells, the blockade of inhibitory receptors on NK cells also demonstrates promise, and several NK cell inhibitory receptors have been explored for their therapeutic potential and clinical utilization.^145–147^ The killer immunoglobulin receptor (KIR) family and CD94/NKG2A heterodimer are the main inhibitory receptors on human NK cells,^145^ and antibodies targeting KIRs either alone or in combination with other therapeutic agents can enhance the antitumor activity of NK cells.^146^ In addition, antibodies targeting NKG2A also show effectiveness in triggering NK cell responses,^147^ and monalizumab, a novel anti-NKG2A antibody, is currently being evaluated for its antitumor efficacy in clinical trials. Importantly, in addition to inhibitory receptors, activating receptors could also be harnessed, such as by administering cytokines to upregulate their expression or by delivering antibodies coating target cells to elicit NK cytotoxicity.^145^ As an encouraging example of such approaches, Andrade et al. designed antibodies to prevent human cancer cells from losing cell surface MICA and MICB, both stress-induced molecules recognized by activating receptors of NKG2D on NK cells, and found that these antibodies inhibited tumor growth through augmented antitumor immunity mediated mainly by NK cells.^144^ Taken together, harnessing NK cells for therapeutic purposes is a promising option and deserves further exploration.

### Myeloid cells

Myeloid lineage cells encompass heterogeneous cell populations, including granulocytes and mononuclear phagocytes,^148–150^ and have been shown to play critical roles in tumor immunity.^149^

Although neutrophils, the most common subtype of granulocytes, typically function in innate protection against bacterial and fungal infections, their roles in tumor immunity remain controversial.^151^ Szczerba et al. showed that neutrophils escorted circulating tumor cells (CTCs) within the bloodstream and facilitated the metastatic potential of CTCs,^152^ while Ponzetta et al. found that neutrophils were essential for the polarization of a subset of unconventional T cells with an innate-like phenotype, and thus benefited antitumor immunity.^153^ In addition, Fridlender et al. discovered that neutrophils had different states of activation in the TME, with the N1 phenotype taking an antitumorigenic function and the N2 phenotype taking a protumorigenic function.^154^ These findings together underline the functional diversity of tumor-associated neutrophils (TANs). Further investigations are warranted for the comprehensive dissection of TANs in human cancers, which might open new opportunities for regulating neutrophils as a mode of cancer therapy.

Mononuclear phagocytes, composed of monocytes, macrophages, and DCs, function in innate immunity by pathogen sensing and phagocytosis^148^ and serve as interacting cellular components of adaptive immunity by presenting antigens to T cells.^148^ DCs are the key APCs, and two major DC subsets, plasmacytoid DCs (pDCs), and classical or conventional DCs (cDCs), have been identified.^155^ pDCs are capable of producing high levels of type I interferon and play an important role in modulating innate and adaptive immunity.^156^ Although pDCs were originally recognized for their roles in antiviral immunity, recent interest has turned to their contribution to tumorigenesis. The secretory products of pDCs, especially type I IFN, were reported to have both immunogenic and tolerogenic functions in tumor immunity.^157,158^ These cytokines contribute to an immunostimulatory TME by promoting the maturation and activation of DCs and proinflammatory macrophages, by increasing the cytotoxicity of NK and T cells, and by facilitating the differentiation of activated B cells into plasma cells;^52,159,160^ they also drive an immunosuppressive TME by recruiting Tregs or by inducing the expression of immunomodulatory molecules such as those involved in negative regulatory pathways.^158,160,161^ In addition, pDCs can act as professional APCs to regulate antitumor immune responses.^157^ Thus, the complex roles of pDCs in tumor immunity remain elusive. cDCs consist of two subtypes, described as cDC1s and cDC2s, which demonstrate different phenotypes, functions, and transcriptional factor dependencies.^162^ cDC1s are specialized in presenting antigens on MHC class I molecules to CD8 T cells, while cDC2s are proficient in presenting antigens on MHC class II molecules to CD4 T cells.^163^ cDC1s contribute to antitumor immunity through local effects within the TME and by antigen delivery to tumor-draining lymph nodes (dLNs). Specifically, cDC1s in the TME secrete chemokines such as CXCL9 and CXCL10 to facilitate the recruitment of effector T cells and NK cells into tumors, and they produce cytokines to help maintain the cytotoxic functions of effector cells.^164^ In addition, cDC1s can migrate to dLNs and deliver tumor antigenic peptides to naïve CD8 T cells, leading to the activation and initiation of antigen-specific immune responses.^165^ cDC2s are the most frequent and highly heterogeneous DC subset, and they promote a wide range of CD4 T-cell-mediated immune responses.^166–168^ Although they are assumed to function mainly through activating CD4 T cells, the exact function of cDC2s in antitumor immunity remains elusive, and growing interest has been directed toward deciphering the heterogeneity and function of cDC2s in the TME.

Macrophages are phagocytic cells, comprising a heterogeneous population with complex phenotypic and functional properties in the TME. Macrophages can eliminate malignant cells through phagocytosis or through producing soluble factors to induce tumor cell apoptosis.^169^ In addition to the direct tumor-killing capability, macrophages play important roles in modulating tumor progression through mechanisms such as angiogenesis, fibrosis, and immunosurveillance. Macrophages could regulate angiogenesis in the TME by secreting different molecules to mobilize or neutralize vascular endothelial growth factor to exert proangiogenic or antiangiogenic functions.^170^ Similarly, macrophages are also crucial orchestrators of tumor-associated fibrosis through different mediators to promote or inhibit extracellular matrix accumulation and to alter the phenotype of neighboring fibroblasts.^171,172^ The induced fibrosis could regulate the infiltration and activation of T cells. In addition, macrophages can mediate T-cell activation through cell interaction by producing IL-12 and by expressing costimulatory molecules including CD86, whereas they can mediate T-cell suppression by expressing T-cell inhibitory molecules, by secreting immunosuppressive cytokines or by promoting the recruitment of immunosuppressive Tregs.^72^ Thus, tumor-associated macrophages (TAMs) have profound effects on the TME and may offer new opportunities for cancer immunotherapy.

### Novel technologies to dissect tumor-infiltrating immune cells at the single-cell level

As the key component of adaptive immunity, T cells have been the core pillar of immunotherapy owing to their specificity for antigen recognition and their potent tumor-killing ability.^96^ TILs are a heterogeneous population composed of diverse subsets (e.g., CD8^+^, CD4^+^ T_H_1, T_H_2, T_H_17, and Tregs) with complex functional states (e.g., naive, effector, memory, and dysfunctional).^101^ In addition, these T-cell subsets exhibit a preference for tissue distribution^173,174^ and demonstrate dynamic properties such as cross-tissue migration and state transitions in the TME.^174^

**Fig. 2:**
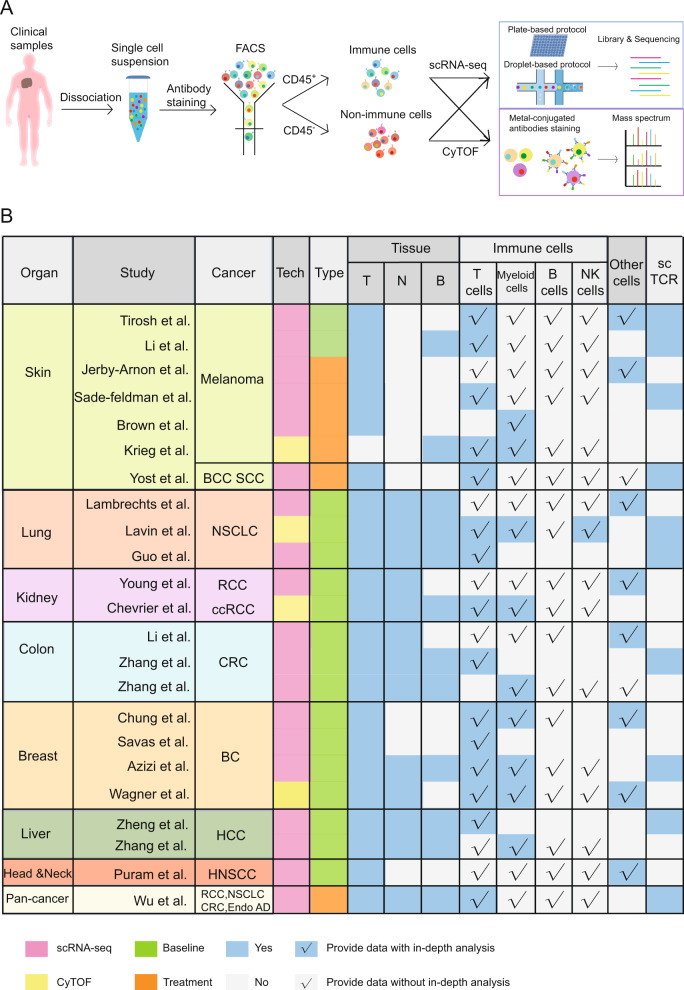
Single-cell profiling strategies and single-cell studies performed in different cancer types. **a** The workflow of single-cell technologies, including scRNA-seq and CyTOF. For scRNA-seq, plate-based and droplet-based strategies are commonly used for in-depth or large-scale analyses. **b** The summary of current single-cell-based studies for the dissection of immune infiltrates in various tissues and cancer types. Tech technology, FACS fluorescence-activated cell sorting, T tumor, N adjacent normal or healthy tissues, P peripheral blood, Other cells, nonimmune cells, including malignant cells and stroma cells, scTCR single-cell TCR information, BCC basal cell carcinoma, SCC squamous cell carcinoma, NSCLC non-small cell lung cancer, RCC renal cell carcinoma, ccRCC clear cell renal cell carcinoma, CRC colorectal cancer, BC breast cancer, HCC hepatocellular carcinoma, HNSCC head and neck squamous cell carcinoma, Endo AD endometrial adenocarcinoma

**Fig. 3:**
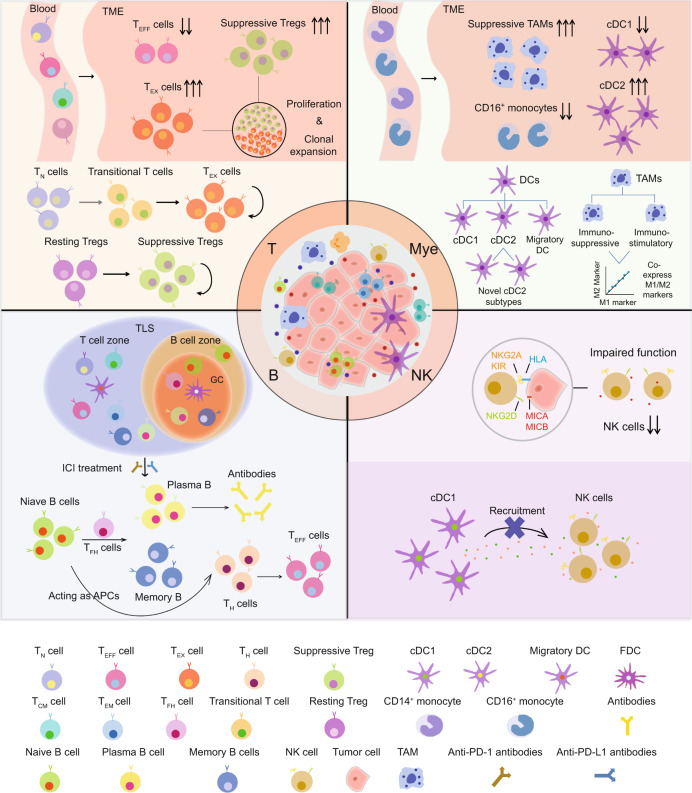
Functional properties and dynamic changes of immune cells in the tumor microenvironment. T cells in peripheral blood infiltrate into tumors and undergo functional state transitions, possibly driven by the immunosuppressive microenvironment. Naive CD8 T cells or CD4 T_H_ cells differentiate into transitional states and finally reach exhausted states, while resting Tregs transit into suppressive states in tumors.^179,181^ Such state transitions result in a reduction of effector T cells yet an accumulation of exhausted T cells and suppressive Tregs, both of which are proven to be proliferating and highly clonally expanded in the TME.^174,179^ Myeloid cells in blood are mainly monocytes, including CD14^+^ and CD16^+^ subsets, while these cells tend to differentiate into macrophages and DCs in tumors.^173,206^ The TME sculpts them to harbor immunosuppressive phenotypes, resulting in an accumulation of suppressive TAMs and cDC2s but a reduction of CD16^+^ monocytes and cDC1s.^173,184^ In addition, single-cell interrogation facilitates the identification of novel subsets of cDCs and TAMs in the TME and reveals that TAM subtypes tend to coexpress M1 and M2 signatures, thus inconsistent with the polarization models.^167,190,191^ NK cells exert cytotoxic functions with perforin and granzymes when activated by the integrated signals of activating and inhibitory receptors,^138–140^ yet they show reduced cell numbers, impaired cytotoxic function and an impeded orchestrating effect for immune responses exemplified by the hampered cDC1 recruitment in the TME.^140,173^ The functional defects of NK cells are possibly driven by tumor cells through secreting immunosuppressive factors and expressing ligands of inhibitory receptors while decreasing the expression of ligands of activating receptors to hinder NK activation.^139,140,173^ B cells play important roles in antitumor immunity and ICI treatment, as B cells and TLSs, containing aggregates of immune cells, including T cells, B cells and FDCs, are found to mediate improved responses to ICIs, the mechanism of which involves the activation of T_FH_ and B cells.^125,134,136^ The activated B cells can differentiate not only into plasma B cells to produce antibodies to clear cancer cells but also into active T-cell-mediated immune responses by presenting antigens to CD4 T_H_ cells that could promote the activation of CD8 T cells.^134^ T_N_ cell naive T-cell, T_CM_ cell central memory T-cell, T_EM_ cell effector memory T-cell, T_H_ cell T helper cell, T_FH_ cell T follicular helper cell, T_EFF_ cell effector T-cell, T_EX_ cell exhausted T-cell, Tregs regulatory T cells, TAMs tumor-associated macrophages, DCs dendritic cells, cDC classical dendritic cell, FDC follicular dendritic cell, GC germinal center, TLS tertiary lymphatic structure, ICIs immune checkpoint inhibitors, TME tumor microenvironment, NK natural killer, APCs antigen-presenting cells, HLA human leukocyte antigen, Mye myeloid cell

Because of such heterogeneity, conventional approaches are unable to dissect the features of diverse T-cell populations. With recent advances in single-cell technologies, large-scale characterization of tumor-infiltrating immune cells at single-cell resolution is of tremendous interest to cancer immunologists. Recently, innovative single-cell approaches, including mass cytometry and single-cell RNA sequencing (scRNA-seq), have gained momentum and have driven vital biological insights into the properties of tumor-associated T cells and other immune cells (Figs. 2 and 3).

Single-cell protein analysis is a pivotal approach to understanding the phenotypic heterogeneity of TILs. Mass cytometry, or cytometry by time-of-flight (CyTOF), utilizing metal-isotope-labeled antibodies in combination with finely tuned mass spectrometry-based detection, enables simultaneous quantification of more than 40 proteins from millions of individual cells at low cost.^175^ By contrast, scRNA-seq, including plate-based and droplet-based strategies^27^ (Fig. 2a), can quantify thousands of transcripts simultaneously; thus, it is able to reveal rare cell populations, uncover complex regulatory mechanisms, and track the developmental trajectories of distinct cell lineages. Recently, these two approaches have been applied to assess the tumor ecosystems of various cancer types (Fig. 2), as both proteomes and transcriptomes could provide important insights into the functional features of the immune infiltrates in the TME.

### Capturing T-cell states by single-cell technologies

Single-cell studies have been performed to delineate T-cell characteristics such as compositions, functional states, and dynamic changes in tumor lesions of different cancer types involving various tissues, such as skin, lung, kidney, breast, colon, head and neck, and liver^27,176^ (Fig. 2b). Such studies provide a glimpse into immune infiltrates in tumors, offering opportunities to understand the mechanisms of immune evasion and to develop novel strategies to further reinforce antitumor immunity.

Melanoma, typically harboring a high tumor mutational burden (TMB),^177^ is on the leading edge of tumor immunology research due to its high response rate to ICI therapies.^178^ Tirosh et al. applied the scRNA-seq approach to investigate the multicellular ecosystem of metastatic melanomas and profiled the phenotypic diversities of malignant cells and nonmalignant cells.^104^ Although limited by cell numbers, T-cell analyses of this study recovered the exhaustion phenotype of TILs. Likewise, Li et al. discovered that dysfunctional TILs were a highly proliferating, clonal, and dynamically differentiating cell population that exhibited a continuous differentiation spectrum within the TME of melanoma.^179^ Such analyses provide an opportunity for us to understand the T-cell characteristics in the TME and expand our knowledge about T-cell exhaustion in human melanoma.

Lung cancer also harbors extensive genomic alterations and has a better response to checkpoint blockade therapies;^80,177^ thus, it is commonly targeted in cancer immunotherapy. Lambrechts et al. presented a single-cell transcriptomic catalog of the tumor ecosystem in human lung cancer.^180^ However, despite the large number of immune cells, they underlined the phenotypic molding of stromal cells and their regulation of immune cells, thus establishing indirect connections with immunotherapy. In contrast, Lavin et al. utilized mass cytometry to provide an immune cell atlas associated with early-stage lung cancer.^173^ They uncovered an immunosuppressive microenvironment by observing a significant reduction in CD8 effector T cells, accompanied by the expansion of Tregs and exhausted T cells at the tumor sites. Similarly, Guo et al. depicted the transcriptomic landscape of T cells in NSCLC with scRNA-seq.^181^ They portrayed the developmental trajectory of TILs and identified two clusters of CD8 T cells exhibiting functional states preceding exhaustion, both of which were associated with good prognosis. These findings provide deeper insights into the functional states and dynamics of T cells in lung cancer that will be helpful in cancer treatment and patient stratification.

RCC is the most common kind of kidney cancer in adults and harbors a high prevalence of insertion and deletion mutations,^182^ albeit with a lower TMB than melanoma or NSCLC.^177^ Subsets of RCC patients could benefit from ICI treatment, and nivolumab, an anti-PD-1 antibody, has been approved for the treatment of metastatic RCC.^21^ Young et al. profiled the single-cell transcriptomes of healthy and cancerous human kidneys and depicted the cellular identities and compositions of renal tumors.^183^ Although they identified both immune and nonimmune cells, they focused on the nonimmune compartment yet provided limited information about immune cell functions in RCC. In contrast, Chevrier et al. presented a mass-cytometry-based single-cell atlas of immune infiltrates in clear cell RCC (ccRCC), the most common type of RCC, and revealed the phenotypic complexity of immune cells in the TME.^184^ T cells illustrated immunosuppressive phenotypes, including functionally exhausted T cells and suppressive Tregs, in ccRCC. These observations expand our view on the phenotypic diversities of T cells and provide candidate targets for immunotherapy in RCC.

Colorectal cancer (CRC) is compelling for immuno-oncologists because tumor-infiltrating immune cells were found to be better predictors for CRC patient survival than histopathological methods.^95^ In addition, ICIs are effective in CRC patients with microsatellite instability (MSI) but not in microsatellite stable patients,^185^ the molecular underpinnings of which remain elusive. Li et al. performed transcriptome profiling of CRC tumor ecosystems using scRNA-seq.^186^ This study provided limited biological insights, especially for immune cell functions in CRC, due to its focus on clustering algorithm development. Notably, Zhang et al. performed comprehensive analyses of T cells in CRC with integrated single T-cell analysis by the RNA sequencing and TCR tracking (STARTRAC) framework.^174^ They delineated the dynamic relationships of diverse T-cell subsets with distinct functions and clonalities. In addition, they revealed one Th1-like subset preferentially enriched in MSI patients, suggesting the underlying cellular mechanisms for their favorable responses to ICIs. These findings deepen our understanding of T-cell features in CRC tumors and accelerate the dissection of mechanisms of ICI treatment.

Although breast cancer (BC) has historically been regarded as difficult to treat with immunotherapy due to its immunologically “cold” phenotype,^187^ recent studies suggest that ICIs have the potential to improve outcomes of subsets of BC patients.^23^ Single-cell studies provide a glimpse into the tumor ecosystem, including immune cell diversity in BC. Chung et al. performed single-cell transcriptome profiling in primary BC.^188^ Although limited by cell numbers, this study uncovered intratumoral heterogeneity and observed that T cells displayed immunosuppressive characteristics in the TME of BC. Similarly, Savas et al. deciphered the cellular heterogeneity of T-cell subpopulations in BC and found that a tissue-resident memory T cell subset expressing high levels of immune checkpoint molecules and effector proteins contributed to BC immunosurveillance.^189^ Although such analyses provide important clues for T-cell functions in BC oncology, the phenotypic plasticity and dynamic changes of TILs in BC need further exploration. Importantly, Azizi et al. provided a large-scale single-cell transcriptional map of immune cells in human BC.^190^ They discovered that T-cell clusters were characterized by diverse patterns of environmental signatures, tumor-resident T cells were mapped on the continuous activation and differentiation trajectories, and combinatorial environmental stimuli and TCR utilization shaped the diverse phenotypes of TILs in BC. This study offers a more nuanced view of phenotypic diversity for tumor-infiltrating immune cells in BC, which might facilitate a better understanding of mechanisms of cancer progression and therapeutic responses. Although different BC subtypes were involved, this study, limited by patient numbers, was unable to provide adequate information to assess the distinction of immune cell phenotypes across BC subtypes. In contrast, Wagner et al. deciphered single-cell proteomics of tumor and immune cells in patients with BC subtypes, thus providing an opportunity to portray the ecosystem differences, particularly for immune cell distinctions, among different BC subtypes.^191^ Notably, they observed a higher frequency of Tregs and exhausted T cells in high-grade estrogen receptor-negative (ER^−^) and ER-positive (ER^+^) tumors, probably indicating the cellular basis of better responses to ICIs for the ER^−^ subtype and a subset of high-grade ER^+^ patients. Such tumor–immune relationships in the BC ecosystem could help guide patient stratification and facilitate personalized immunotherapy.

Other human cancers with single-cell characterizations of the TME include head and neck squamous cell carcinoma (HNSCC) and hepatocellular carcinoma (HCC). Puram et al. depicted the primary and metastatic tumor ecosystems in HNSCC by scRNA-seq.^192^ They characterized distinct T-cell subsets and defined a putative T-cell exhaustion program in HNSCC; however, they provided limited information about T-cell functions owing to their focus on the nonimmune compartment. Zheng et al. portrayed the transcriptional profiles of single T cells in HCC and found that intratumoral T cells demonstrated immunosuppressive phenotypes based on the observation of clonal enrichment of infiltrating Tregs and exhausted CD8 T cells in tumor sites. This study is the first large-scale and in-depth analysis of TILs, revealing the underlying cellular mechanisms of HCC progression.^105^

Collectively, these baseline analyses elucidated the basic properties of TILs in various cancer types. TILs in different cancers exhibit both common and specific characteristics in antitumor immunity,^174^ possibly driven by the specialized tissue microenvironment of different organs. Therefore, comprehensive dissection of T-cell features in more cancer patients will shed light on the mechanisms of cancer progression and the differences in therapeutic responses, thus facilitating personalized immunotherapy in cancer treatment.

Although the cellular compositions and functional states are critical properties of TILs, their antigen specificities also serve as pivotal determinants of antitumor immune responses and can affect the efficacies of immunotherapies. Emerging evidence shows that a considerable proportion of T cells in tumors share TCRs with those in adjacent normal tissues, which might indicate their irrelevance to antitumor immunity. Such T cells could represent bystander T cells that target background mutations or viral infections,^193–195^ or they might reflect the continuous migration of effector or memory T cells from blood to tissues, driven by intratumoral inflammatory responses.^174,196^ Importantly, clinically effective TILs are shown to be T cells that target tumor neoantigens;^197^ thus, isolating tumor-reactive T cells is critical for T-cell-based therapies. Indeed, innovative strategies have been established for the identification, isolation, and expansion of neoantigen-specific T cells. Simoni et al. demonstrated that specific markers such as CD39 could be utilized to identify tumor-reactive T cells.^195^ Tran et al. cocultured TILs with autologous DCs transfected with in vitro transcribed mRNAs encoding tandem minigenes that encode short peptides of mutated genes in autologous tumors to isolate T cells specifically reactive with tumor neoantigens.^113^ Furthermore, Dijkstra et al. utilized the coculture of autologous tumor organoids with PBLs to enrich tumor-reactive T cells from peripheral blood.^198^ Such approaches provide efficient means to isolate tumor-reactive T cells for clinical applications.

While the above baseline profiling of treatment-naive tumors provides the intrinsic properties of TILs in diverse cancer types, treatment- or intervention-based studies can offer better opportunities for understanding the molecular underpinnings of immunotherapies and for developing novel approaches to predict clinical efficacies. Jerby-Arnon et al. investigated malignant cell states in human melanoma tumors before and after ICI treatments with scRNA-seq.^199^ They discovered that malignant cells could express a resistance program associated with T-cell exclusion and immune evasion, and the inhibition of such a program in combination with immunotherapy could reduce tumor growth. Such findings suggest a new strategy to overcome ICI resistance. Compared with molecular changes in cancer cells, however, more attention has been paid to the phenotypic and functional dynamics of TILs upon ICI treatments. By profiling single immune cells from melanoma patients treated with ICI,^200^ Sade-Feldman et al. found that two unique states of CD8 T cells expressing TCF7 protein or dysfunctional signatures could predict the success or failure of checkpoint immunotherapies, underlining the clinical significance of heterogeneous T-cell subtypes in the TME. Similarly, Yost et al. performed paired single-cell RNA and TCR sequencing on T cells from patients with basal or squamous cell carcinoma (BCC or SCC) treated with an anti-PD-1 inhibitor and revealed clonal replacement of tumor-specific T cells following PD-1 blockade.^201^ Specifically, they found that ICI treatment induced the clonal expansion of T cells, while the expanded clones did not derive from pre-existing TILs but instead consisted of novel clonotypes. Such observations underscored the significance of systemic immune responses and the necessity of recruiting peripheral T cells for effective ICI treatment.

T cells in the peripheral blood can migrate and infiltrate into tumors to replenish the effector pool; thus, human peripheral blood mononuclear cells are also intensely investigated for their changes before and after ICI treatments. Wu et al. performed single-cell sequencing of the RNA and TCRs of individual T cells in patients with different cancer types and uncovered that the clonal expansion of effector-like T cells at a systematic level across tumors, adjacent normal tissues and peripheral blood could mediate better responses to anti-PD-L1 therapies.^202^ These findings indicate that effective responses to ICIs require replenishment of fresh, nonexhausted T cells from peripheral blood. Likewise, Krieg et al. used mass cytometry to characterize the immune cell subsets in the peripheral blood of patients with metastatic melanoma before and after anti-PD-1 immunotherapy.^203^ They found that T cells in the peripheral blood were reduced while CD8 T cells in tumors were increased in responders, suggesting a higher migratory capacity of CD8 T cells responsible for ICI responses and the importance of systematic immunity. Of note, they discovered that the frequencies of myeloid cells could also predict responsiveness to anti-PD-1 therapies, highlighting the importance of myeloid cells in antitumor immune responses and their potential clinical applications.

### Capturing myeloid cell heterogeneity by single-cell technologies

Although T cells have been the focal target of cancer immunotherapies, myeloid cells have also gained traction in recent years since they exhibit specific phenotypes and functions in the TME that could impact cancer progression and immunotherapy responses either by regulating T-cell functions or by directly regulating tumor cell growth (Fig. 3).

As professional APCs, DCs play a central role in T-cell activation and are necessary for the maintenance of long-lasing antitumor adaptive immune responses. Regarding the phenotypes and functions of different DC subsets, pDCs and cDC1 are composed of a relatively homogeneous population, and thus have well-established functions, although their roles in antitumor immunity might need further verification. By contrast, cDC2s comprise a heterogeneous population, and their functional roles remain elusive due to heterogeneity. Single-cell technologies enable comprehensive dissection of DC compartments in health and disease conditions. For example, with the use of scRNA-seq, Villani et al. identified a new subdivision within the cDC2 subset in human blood.^204^ Similarly, Ductertre et al. revealed functionally distinct subsets of cDC2 based on a combination of several markers and identified circulating inflammatory cDCs that were correlated with disease activity in rheumatic diseases.^205^ Regarding DC subsets in tumors, Brown et al. discovered that two principal cDC2 lineages, characterized by distinct developmental pathways and transcriptional regulators, showed distinct pro- and anti-inflammatory potentials in mouse and human melanoma.^167^ Zhang et al. reported a subset of cDCs expressing *LAMP3*, which can migrate from HCC tumors to hepatic lymph nodes and regulate lymphocyte activation.^206^ These findings highlight the heterogeneity of DC subpopulations and pave the way for the identification of specific DC subset-targeting immunotherapies.

TAMs play multiple roles in tumor development and act as critical regulators of the complex TME. TAMs harbor highly plastic phenotypes and can be induced to polarize toward classically activated (M1) or alternatively activated (M2) phenotypes to exert immunostimulatory or immunosuppressive functions in tumor immunity.^207,208^ M1 macrophages exhibit a proinflammatory phenotype with tumor-killing capability, while M2 macrophages exert immunoregulatory function by promoting immune suppression and tumor progression.^207,209^ Several studies have proposed that macrophages in tumors shift toward the M2 phenotype and produce anti-inflammatory cytokines.^188,210^ However, emerging studies powered by single-cell technologies showed that TAM behavior did not comport with the polarization model in the TME.^184,211^ Azizi et al. found that M1 and M2 signatures were positively correlated in myeloid populations in human BC,^190^ and Wagner et al. also confirmed the coexpression of pro- and anti-inflammatory markers in TAM populations, albeit with the immunosuppressive TME in BC.^191^ Notably, using single-cell technologies, researchers have discovered specific functional characteristics of myeloid cells in tumors. Lavin et al. identified TAM subsets that could compromise T-cell-mediated antitumor immunity and revealed the enrichment of immunosuppressive macrophages in the TME of early-stage lung cancer.^173^ Akin to such observations, Chevrier et al. identified an immunosuppressive TAM subset that expressed both pro- and anti-inflammatory markers and revealed its association with exhausted T cells and Tregs in ccRCC.^184^ Zhang et al. leveraged scRNA-seq to generate an atlas of immune and nonimmune cells in CRC patients.^212^ They identified two distinct TAM subsets characterized by inflammatory and angiogenic signatures, respectively, which showed differential sensitivity to CSF1R blockade. Importantly, they suggested that the depletion of specific TAM subsets could contribute to the improvement of myeloid-targeted immunotherapies and could achieve synergistic effects when combined with ICB therapies.^212^ These discoveries uncover the interplay between innate and adaptive immunity and indicate that TAMs should be considered a new class of targets for immunotherapies.

## Concluding remarks

Cancer immunotherapy, despite its long history, has blossomed into fruition only in recent years with the advances of multiple forms of treatment, including cancer vaccines, ACT and ICIs. A systematic review of the landmark studies in the progress of cancer immunotherapy could facilitate a better understanding of the basic principles, advantages and limitations of various types of immunotherapies, and thus will help promote the development of novel strategies to circumvent their drawbacks and achieve optimal clinical efficacy.

Despite impressive advances in immunotherapies, obstacles, and challenges, including limited response rates, the inability to predict clinical efficacy, and potential side effects such as autoimmune reactions or cytokine release syndromes, remain and hinder the further application of immunotherapies in clinics.^213^ Tumor-infiltrating immune cells, in particular T cells, serve as the cellular underpinnings of cancer immunotherapies, and a better understanding of immune cells in the TME is essential for deciphering mechanisms of immunotherapies, defining predictive biomarkers, and identifying novel therapeutic targets. Although the heterogeneous cell populations in the TME stand out as the key barrier to delineate the tumor ecosystems, the advances in single-cell technologies, in particular scRNA-seq and CyTOF, have fostered the explosion of single immune cell characterizations. T cells have been the focus of such analyses, and significant biological insights have been obtained about the engagement of T-cell phenotypic and functional diversity in cancer immunotherapies. A systematic overview of the characteristics of TILs in different cancers would shed light on the distinctive mechanisms of immune responses; thus, a more comprehensive pan-cancer analysis of TILs is warranted to elucidate the differences in responses among different cancer types.

The TME is a complex ecosystem that contains heterogeneous populations of cancer cells, stromal cells and immune cells. Crosstalk among these cell types could remodel the TME and regulate cancer progression. Notably, immune cells in tumors act in concert to control tumor growth, and the efficacies of immunotherapies are dependent on the orchestrated responses of both innate and adaptive immune cells.^214^ Although T cells play a crucial role in cancer immunotherapies, the sophisticated cellular interplay and communication in the complex ecosystem of the TME suggest the importance of other immune cells, including myeloid cells, NK cells and B cells, in antitumor immunity.^215^ Indeed, recent single-cell studies have underlined the immunoregulatory roles of myeloid cells in human tumors, providing novel insights into the functional states and developmental lineages of heterogeneous myeloid subsets and their connections with immunotherapies.^173,184,190^ NK cells act as complementary effector cells to antitumor immunity compared with T cells, and thus have important applications for cancer treatment. Importantly, NK cell activation involves the integrated signaling of activating and inhibitory molecules, which present attractive targets due to their functional similarities with costimulatory and coinhibitory molecules in T cells.^216^ A growing appreciation also emerges for the roles of B cells due to their diverse functions, including antibody production, cytokine secretion, antigen presentation, and lymphoid architecture organization, in antitumor immunity. Recent studies have highlighted the vital roles of B cells in cancer treatment and their involvement in immunotherapies, although the precise mechanisms need further investigations.^125,133,136^

Although the cell–cell interactions in the TME have been elucidated through the expression of ligand and receptor pairs in recent single-cell studies,^217,218^ which seem sensible but require further experimental validation, accumulating evidence indicates that cell–cell interactions occur in distinct spatial regions or local tissue niches in tumors.^196,219^ Moncada et al. introduced multimodal intersection analysis for the identification and spatial mapping of distinct populations within heterogeneous tissues and found that cellular components of the TME showed spatially restricted enrichments, and certain cell types exhibited distinct coenrichments to coordinately regulate tumor progression.^219^ Notably, antitumor immune responses have also been proven to exhibit spatial heterogeneity.^220,221^ Reuben et al. observed extensive spatial differences in T-cell density and clonality in distinct regions of the same tumors, suggesting substantial intratumoral heterogeneity of the T-cell repertoire.^222^ Consistently, Losic et al. demonstrated spatial cancer–immune interactions and found that tumor-associated immune infiltrates exhibited regional heterogeneity with distinct tumor regions showing different levels of immune clonal expansion and antigen-specific T-cell responses.^221^

The spatial heterogeneity of antitumor immunity may correlate with the intratumoral heterogeneity of cancer cells. Cancer cells are commonly composed of different subclones located in distinct compartmentalized regions that harbor distinct genomic, phenotypic, and antigenic diversities.^223,224^ The distinct tumor regions contain locally generated neoantigens that could be perceived by the immune system to elicit spatially restricted immune responses, thus shaping the spatial landscape of antitumor immunity.^196,223,225,226^ Such relevance could be further confirmed by the discovery that the intratumoral TCR repertoire can be classified into expanded ubiquitous and regional TCRs that correspond to the tumor mutational landscape.^196^ Overall, these discoveries provide insight into the significant association between spatially constrained immunological heterogeneity and intratumoral genomic heterogeneity and highlight the importance of deciphering the complex spatial compositions and organizations of cellular components in the TME at the single-cell level.

Although scRNA-seq enables the systematic characterization of cell populations in the TME, the spatial organizations and spatial gene expression patterns of tumor-associated immune cells remain poorly understood. Many technologies that aim to characterize spatial proteomics or spatial transcriptomics using imaging or sequencing methods based on cells in a specific spatial context are currently being developed to provide spatial information on the cellular and molecular components of heterogeneous tissues. Multiplexed imaging methods for spatial proteomics detection include immunofluorescence-based approaches, such as cyclic immunofluorescence, which utilizes an iterative process of repeatedly collecting low-plex fluorescence images, and then assembling them into a high-dimensional representation,^227,228^ and epitope-based methods, such as multiplexed ion beam imaging and imaging mass cytometry, both of which utilize a mass spectrometer for readout and enable the simultaneous detection of up to 40 metal-labeled antibodies at subcellular resolution.^229,230^ Spatial transcriptomics quantification is now an active research field for cancer investigation. Fluorescence in situ hybridization (FISH) is the main approach to determine the spatial location and abundance of RNA molecules in their native cellular environment. Based on FISH, improved methods, such as single-molecule RNA imaging approaches, including single-molecule RNA FISH, sequential FISH, and multiplexed error-robust FISH,^231–234^ have been developed to decipher the spatial information of the cellular mRNA content within tissues, although they have different performances regarding spatial resolution, quantitative accuracy, and the number of features that can be profiled. In addition to imaging technologies, sequencing techniques are also involved in the spatial quantification of RNA molecules. In situ sequencing,^235^ especially fluorescent in situ RNA sequencing, enables in situ profiling of the transcriptome in fixed cells.^236^ Furthermore, transcriptome in vivo analysis allows capturing mRNA from live single cells in their natural microenvironment.^237^ With advances in spatial quantification methods, single-cell spatial profiles of tumor-associated immune cells will reveal novel parameters that are crucial for antitumor immunity.

Understanding the orchestrated organizations and interactions of cancer and immune cells in a spatial coordinate system will provide further insights into cancer progression and could provide clues for improving the efficiency of current immunotherapies. As further technologies emerge in the single-cell field, especially for the improvements in spatial mapping and quantification approaches, we anticipate that the systematic and comprehensive interrogation of in situ crosstalk of different immune cells and cancer cells in the TME, as well as their dynamic changes upon treatment, will be achieved, and such advances will further propel the clinical success of immunotherapies.
